# Quantitative measurement of blood flow in paediatric brain tumours—a comparative study of dynamic susceptibility contrast and multi time-point arterial spin labelled MRI

**DOI:** 10.1259/bjr.20150624

**Published:** 2016-06

**Authors:** Rishma Vidyasagar, Laurence Abernethy, Barry Pizer, Shivaram Avula, Laura M Parkes

**Affiliations:** ^1^ Florey Institute of Neuroscience and Mental Health, Heidelberg, Melbourne, VIC, Australia; ^2^ Department of Anatomy and Neuroscience, University of Melbourne, Melbourne, VIC, Australia; ^3^ Department of Radiology, Alder Hey Children's NHS Foundation Trust, Liverpool, UK; ^4^ Department of Paediatric Oncology, Alder Hey Children's NHS Foundation Trust, Liverpool, UK; ^5^ Centre for Imaging Sciences, Faculty of Medicine and Human Sciences, University of Manchester, Manchester, UK

## Abstract

**Objective:**

Arterial spin-labelling (ASL) MRI uses intrinsic blood water to quantify the cerebral blood flow (CBF), removing the need for the injection of a gadolinium-based contrast agent used for conventional perfusion imaging such as dynamic susceptibility contrast (DSC). Owing to the non-invasive nature of the technique, ASL is an attractive option for use in paediatric patients. This work compared DSC and multi-timepoint ASL measures of CBF in paediatric brain tumours.

**Methods:**

Patients (*n* = 23; 20 low-grade tumours and 3 high-grade tumours) had DSC and multi-timepoint ASL with and without vascular crushers (VC). VC removes the contribution from larger vessel blood flow. Mean perfusion metrics were extracted from control and *T*_1_-enhanced tumour regions of interest (ROIs): arterial arrival time (AAT) and CBF from the ASL images with and without VC, relative cerebral blood flow (rCBF), relative cerebral blood volume, delay time (DT) and mean transit time (MTT) from the DSC images.

**Results:**

Significant correlations existed for: AAT and DT (*r* = 0.77, *p* = 0.0002) and CBF and rCBF (*r* = 0.56, *p* = 0.02) in control ROIs for ASL-noVC. No significant correlations existed between DSC and ASL measures in the tumour region. Significant differences between control and tumour ROI were found for MTT (*p* < 0.001) and rCBF (*p* < 0.005) measures.

**Conclusion:**

Significant correlations between ASL-noVC and DSC measures in the normal brain suggest that DSC is most sensitive to macrovascular blood flow. The absence of significant correlations within the tumour ROI suggests that ASL is sensitive to different physiological mechanisms compared with DSC measures.

**Advances in knowledge:**

ASL provides information which is comparable with that of DSC in healthy tissues, but appears to reflect a different physiology in tumour tissues.

## INTRODUCTION

Central nervous system tumours are the most common group of solid tumours in childhood and comprise 20–25% of all childhood neoplasms. There are many different types with variable survival rates depending on the histology, biology, age and degree of spread. Although some types of brain tumours may be completely resected, many cannot be completely removed without serious morbidity. In addition to surgery, chemotherapy and radiotherapy are also used as adjuvant treatments. Survivors of central nervous system tumours are frequently susceptible to severe and sometimes devastating neuropsychological, neurological and other pathologies, which may be related to factors such as the tumour location and therapy used. Brain imaging is fundamental to the management of brain tumours, and there is a clear need for the development of improved non-invasive measures of tumour characteristics. The potential benefits of using a non-invasive method to monitor paediatric brain tumour (PBT) progression and response to treatment are reduced distress to patients and increased frequency of monitoring, which may improve treatment plans and possibly lead to better patient outcomes.

MRI has long been established as a useful tool for the assessment of PBT.^1^ Currently, dynamic susceptibility contrast (DSC) MRI is used clinically, providing quantitative maps of relative cerebral blood flow (rCBF), relative cerebral blood volume (rCBV) and mean transit time (MTT). This method allows for the identification of areas of contrast agent leakage within a tumour and can differentiate between different tissue characteristics such as cystic regions. Whilst it benefits from a high signal-to-noise ratio, DSC does suffer from some limitations such as absolute quantification of blood flow. Accurate quantification of DSC measures depends both on the method of contrast administration and the assumption that the tracer remains intravascular, which may be invalid in tumours with a compromised blood–brain barrier.^2^

An additional complication in DSC blood flow quantification is the requirement of an arterial input function (AIF), usually chosen from a large vessel. Owing to the differences in blood flow dynamics between adult and paediatric patients, modelling the AIF in a paediatric population can be problematic.^3^ Repeated injections of gadolinium contrast agents are contraindicated in children, and gadolinium agents should not be used at all in very young infants and in children with renal impairment, owing to the risk of nephrogenic systemic fibrosis.^2^ In a research setting, a technique that avoids the distress caused by i.v. injections is much more acceptable to children and parents. Taking these factors into consideration, there is a real need for investigating alternative non-invasive measures of blood flow in PBT.

Arterial spin labelling (ASL) is an MR technique that allows for the non-invasive absolute quantification of CBF, by using blood water as an endogenous tracer. A key benefit of this technique is that it is not reliant on the acquisition of an appropriate AIF, and quantification is much less sensitive to blood–brain barrier (BBB) leakage. This technique, predominately used in research,^4^ has seen significant technical developments in the last few years and a push for clinical use.^5^ The use of ASL in a paediatric population is now starting to gain momentum.^1,6^ Sequence modifications, such as a Look–Locker readout allowing multi-timepoint measurements,^7^ provide an opportunity for more accurate quantification, taking into account different haemodynamics that may be present in children and in tumours in particular.^8^ Multi-timepoint ASL allows us to quantify arterial arrival time (AAT) in addition to cerebral blood flow (CBF). This may be important in the case of neuropathologies such as stroke^9^ and PBT,^8^ where pathology may lead to blood arrival delays. It is also possible to increase the sensitivity of ASL to the microvasculature through the use of magnetic field gradients which cause dephasing of fast-flowing blood [so-called vascular crushers (VC)].^10^ VC reduce the contribution of signal from the large vessels and so allow a more accurate measurement of true capillary blood flow, which is more closely related to tissue metabolism.

This work aimed to compare output measures from DSC to ASL, with and without VC in PBTs. The first aim was to compare the sensitivity of each measure to the presence of tumour tissue. Further, we hypothesized that corresponding DSC and ASL measurements would be correlated; specifically: (i) AAT and Delay time (DT) would be significantly correlated in the tumour tissue and healthy tissue, (ii) CBF and rCBF would be significantly correlated in the tumour tissue and healthy tissue and (iii) the correlations would be stronger for ASL measures without VC (i.e. noVC), which are more akin to DSC measures. It is also expected that ASL with VC would provide qualitatively clearer images of altered tumour blood flow and AAT, without contamination from large vessels. This work was designed to provide the first step towards establishing multi-timepoint ASL as a possible clinical tool in PBTs.

## METHODS AND MATERIALS

### Participants

All patient data came from the Alder Hey Children's Hospital, Liverpool. Data were collected from a total of 23 paediatric patients (mean age: 8 years, range: 2–20 years; 11 females and 12 males) with brain tumours on first presentation and 1 intraoperative case as outlined in Table 1. Children under the age of 5 years were generally scanned under general anaesthetic; however, older children were scanned without sedation or anaesthesia. Patients who had undergone previous tumour treatment were excluded from this study.

**Table 1.** Descriptive statistics for output measures from dynamic susceptibility contrast (DSC) and arterial spin labelling (ASL) within both tumour and healthy tissue regions of interest (ROIs). Tumours have been categorized according to tumour types across all samples

Tumour classification	Number of patients (N)	DSC			ASL			ASL VC	
		DT (ms)	MTT (ms)	rCBV (ml/1000 ml)	rCBF (ml/100 ml min^−1^)	CBF (ml/100 ml min^−1^)	AAT (ms)	CBF (ml/100ml min^−1^)	AAT (ms)
Low grade (grades 1 and 2)									
Gliomas	18	130 (SD: 148)	317 (SD: 179)	140 (SD: 139)	167 (SD: 172)	87 (SD: 83)	819 (SD: 272)	52 (SD: 44)	1086 (SD: 315)
Choroid plexus papilloma	1	91	1457	327 (SD: 126)	161	72	346	43	626
Pineal germ-cell tumour	1	212	712	83 (SD: 100)	66	28	1367	No data	No data
High grade (3 and 4)									
Medulloblastoma	2	196 (SD: 23)	572 (SD: 265)	550 (SD: 25)	36	111 (SD: 16)	655 (SD: 131)	55 (SD: 4)	920 (SD: 13)
PNET	1	275	938	161.09	96	43	935	54	1178
Anaplastic astrocytoma (intraoperative example)	1	119	633	91	104	104	826	111	866
Healthy tissue									
Control ROI from the group of patients with only glioma	18	143 (SD: 179)	497 (SD: 94)	244 (SD: 377)	261 (SD: 293)	75 (SD: 54)	811 (SD: 288)	40 (SD: 22)	1049 (SD: 319)
*t*-test between control and tumour ROI from the group of patients with only glioma (*p*-value)	18	0.6	0.0009	0.268	0.001	0.6	0.9	0.1	0.7

AAT, arterial arrival time; CBF, cerebral blood flow; DT, delay time; MTT, mean transit time; PNET, primitive neuroectodermal tumours; rCBF, relative cerebral blood flow; rCBV, relative cerebral blood volume; SD, standard deviation; VC, vascular crushers.

### MRI protocol

All imaging was carried out on a 3 T Philips Achieva^®^ TX MR scanner (Best, Netherlands). ASL sequences were added to the standard clinical MR protocol. Scans were carried out in the following order: *T*_1_ weighted (without gadolinium), ASL without VC, ASL with VC, DSC, *T*_1_ weighted acquired post-gadolinium (Gadoteric acid, Dotarem^®^; Guerbet, France) injection.

#### *T*_1_ weighted

*T*_1_ weighted [three-dimensional fast low angle shot (FLASH)] scans both pre- and post-gadolinium injection were acquired with the following parameters: repetition time (TR): 8.3 ms, echo time (TE): 3.8 ms, 1-mm isotropic voxels, field of view (FOV) of 240 mm and acquisition duration approximately 5 min. Post-gadolinium *T*_1_ weighted images were used to identify enhancing tumour regions.

#### Dynamic susceptibility contrast

A *T*_2_* weighted echoplanar imaging DSC sequence was used to sample the “first pass” of the contrast agent through the brain vasculature. Sequence parameters were: TR: 1694 ms, TE: 40 ms, voxel size 1.75 × 1.75 × 4 mm, FOV 224 mm, 25 contiguous slices and 40 dynamics. A dynamic pump injection of 1 ml/5 kg with a rate of infusion of 3 ml s^−1^ was administered on the 10th dynamic of the DSC sequence. A loading dose of 1-ml gadolinium was administered prior to the acquisition.

#### Arterial spin labelling

A spin tagging with alternating radiofrequency labelling scheme (STAR)^11^ with a Look–Locker readout^7^ was used with the following parameters: 3 × 3 × 6-mm voxels; FOV 240 mm; 9 slices with 0.6-mm gap; 8 readout pulses at 300, 670, 1040, 1410, 1780, 2150, 2520 and 2890 ms; TR: 4 s, TE 12 ms; flip angle 40^o^; 30 pairs of label and control images; total acquisition time 4 min. A second set of images was collected with the addition of vascular crushing gradients with velocity encoding (VENC) level of 5 cm s^−1^ with the same scanning parameters, except for an increase in the TE to 24 ms. Since full-brain coverage was not possible, slices were positioned to encompass the tumour.

### Data analysis

#### Arterial spin labelling

ASL analysis was carried out using in-house MATLAB (www.mathworks.com) routines. Raw ASL images were qualitatively checked for motion and artefacts before being processed. Control and label images were subtracted and averaged to provide difference images at each timepoint. A single blood compartment model,^12^ adapted for the Look–Locker readout,^13^ was fit to the averaged images on a voxel-by-voxel basis, producing maps of CBF and AAT (without vascular crushing) and CBF with vascular crushing (CBF_VC_) and AAT with vascular crushing (AAT_VC_) (with vascular crushing). This model assumes that labelled water remains in the blood and that no labelled water leaves the voxel, an approach that has been shown to be reasonably accurate.^12^ With these assumptions, the signal in the difference ASL image (control image − label image), ΔM, can be described by:

(1) $$\frac{d\Delta M\left(t\right)}{dt}=-R_{1}\Delta M\left(t\right)+fm_{a}\left(t\right)$$

where *R*_1_ is the apparent longitudinal relaxation rate of blood during the Look–Locker readout and *f* is the CBF. For STAR labelling, the magnetization of the arterial blood, *m*_a_, is given by:

(2) $$m_{\text{a}}\left(t\right)=2{m_{a}}^{0}\alpha \;\exp\left(-tR_{1\text{b}}\right)\;\text{for}\;t>t_{\text{a}}\;\text{and}\;t<t_{\text{a}}+\tau$$

and *m*_a_ = 0 at all other times. Here, *t*_a_ is the arrival time and *τ* is the bolus width. *m*_a_^0^ is the equilibrium magnetization of arterial blood, *α* is the inversion efficiency (assumed to equal 1) and *R*_1b_ is the true *R*_1_ of blood. According to Ref. 7, *R*_1_ = *R*_1b_ − ln(cos*θ*)/TI_2_, where *θ* is the flip angle and TI_2_ is the spacing of the Look–Locker readout. The solution for ΔM is:

(3) $$\Delta M\left(t\right)=\frac{2{fm_{a}}^{0}\alpha }{\Delta R}\exp\left(-tR_{1}\right)\left[\exp\left(t\Delta R\right)-\exp\left(t_{a}\Delta R\right)\right]\;for\;t>t_{a}\;and\;t<t_{a}+\tau$$

(4) $$\Delta M\left(t\right)=\frac{2{fm_{a}}^{0}\alpha }{\Delta R}\exp\left(-tR_{1}\right)\exp\left(t_{a}\Delta R\right)\left[\exp\left(\tau \Delta R\right)-1\right]\;for\;t>t_{a}+\tau$$

where ΔR = *R*_1_ − *R*_1b_. The mean difference signal ΔM was fit on a voxel-wise basis to Equation (3), extracting values for the two free parameters *f* (CBF) and *t*_a_ (AAT), with fixed values for *τ* = 1000 ms,^14^ *T*_1b_ = 1600 ms^15^ (note *R*_1b_ =1/*T*_1b_) and *m*_a_^0^. *m*_a_^0^ was estimated from a whole-brain estimate of *m*_0_ (found by fitting the ASL control images to a saturation recovery curve) divided by the blood–brain partition coefficient *λ* = 0.9.^16^

#### Dynamic susceptibility contrast

DSC analyses were carried out using MIStar^®^—a commercial software (Apollo Medical Imaging Technology, Melbourne, VIC, Australia). MIStar employs a delay- and dispersion-corrected singular value decomposition deconvolution method^17–19^ to create output maps of rCBF, rCBV, MTT and DT. An AIF was chosen manually in a vessel closest to the region of the tumour that would be the most ideal representation of the blood flow dynamics close to the tumour. The chosen AIF is convolved with the tissue contrast-enhancement signal curve, leading to an impulse response function curve. Aspects of the impulse response function curve allow for the calculation of the cerebral blood volume (CBV) (area under the curve), CBF (curve peak) and MTT (CBV/CBF). DT measures were derived from the delay correction as applied by MIStar, with areas of no blood flow deemed to have the largest DT and MTT. As absolute quantification of DSC CBF measures are not possible,^20,21^ measures are reported as rCBF and rCBV.

#### Tumour region of interest

Tumour region of interest (ROI) volume masks were created on the enhancing tissue regions of the post-gadolinium *T*_1_ weighted images by an experienced paediatric neuroradiologist using MRICRO (www.mricro.com). A separate control ROI with the same size as the enhancing tumour ROI was also created for each patient. These control ROIs were ideally located in the contralateral hemisphere for non-midline tumours. When possible, control regions for midline tumours were chosen within the same structure, but excluding the tumour. Otherwise, ROIs were placed in “healthy” tissue areas close to the tumour area. Registration of the output parameter maps from both the DSC and ASL analyses to the *T*_1_ weighted image was carried out using the SPM co-registration toolbox (www.fil.ion.ucl.ac.uk/spm), and mean signals from the ROIs were extracted.

**Figure 1.**
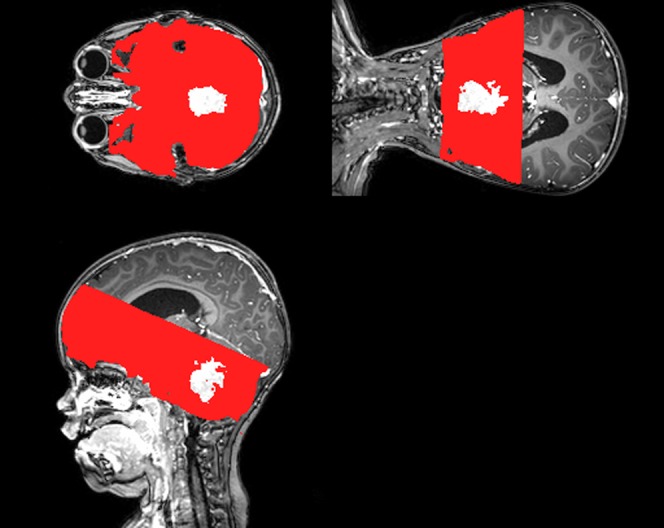
An example of the “global” region of interest (ROI) mask (red) with the tumour ROI subtracted (white region) in a single patient. For colour image see online.

Absolute blood flow values from the ASL and DSC techniques are unlikely to agree owing to a number of global factors affecting both measurements. For ASL, this includes the inversion efficiency, *T*_1_ of blood and equilibrium magnetization of blood and for DSC, differences in tracer concentration and its impact on the relaxivity and differences in small- and large-vessel haematocrit levels. Normalized values, *i.e.* tumour blood flow in relation to global blood flow, should show better agreement. Hence, normalized values were also calculated for the purpose of correlation tests, using a “global” value taken from the entire brain region covered by ASL acquisition, excluding the tumour region (Figure 1). These values were used to normalize both the control ROI and tumour ROI measures. In order to determine the sensitivity of each output measure to the tumour tissue, paired Student's *t*-tests were computed for each measure between the tumour and control region. Pearson correlation tests were computed between the ASL and DSC output measures in both the tumour and control ROIs using MATLAB.

## RESULTS

Table 1 outlines the different tumour types as determined by histopathology. The vast majority of the tumours were identified as low-grade gliomas. Descriptive statistics of perfusion metrics within the tumour ROIs are shown for DSC and ASL with and without vascular crushing, along with data from the control ROI for the group of patients with only glioma. The final row in Table 1 shows the *p-*values from paired Student's *t*-tests between the control and tumour ROIs across the different measures. These paired *t*-tests were carried out across the group of patients with only glioma owing to the larger population size within this group.

No significant differences were noted from the ASL measures (both with and without vascular crushing) in the tumour region when compared with the control region. When considering the measures derived from DSC, MTT and rCBF were both significantly (*p* < 0.001) lower within the tumour region than within the control region; however, rCBV did not show a significant difference.

**Figure 2.**
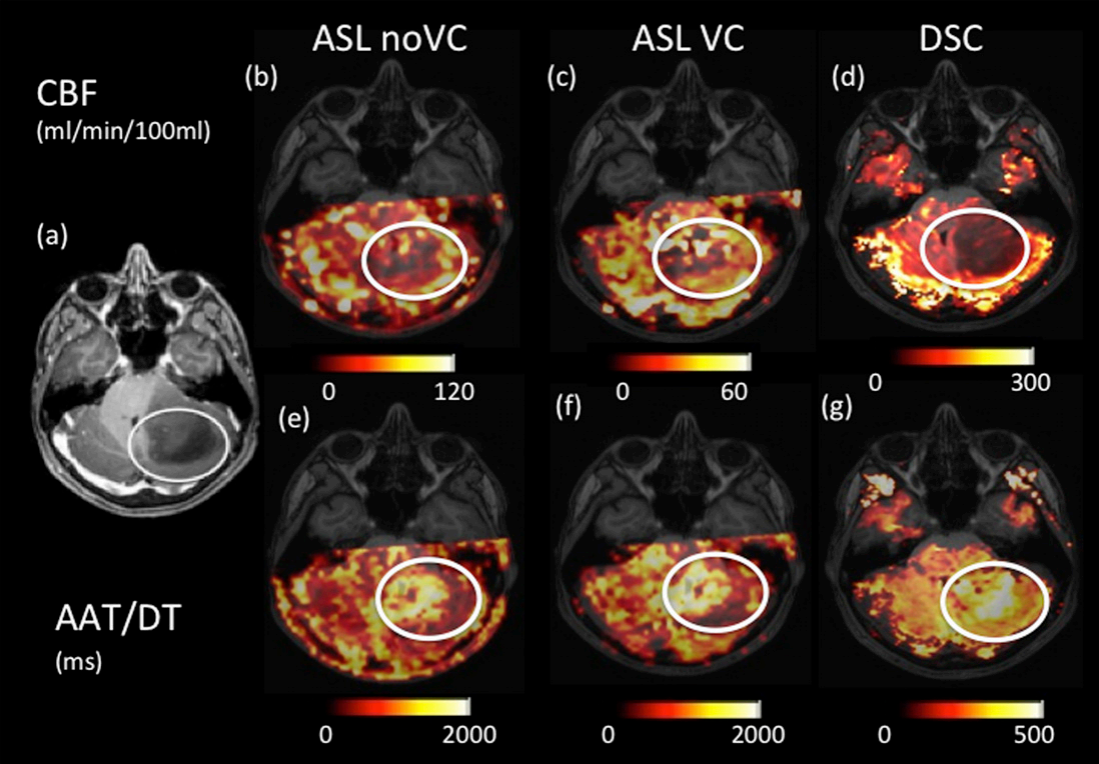
Axial images of a low-enhancing low-grade astrocytoma (highlighted in the white circles). (a) Shows a *T*_1_ weighted post-gadolinium image. Cerebral blood flow (CBF) images for arterial spin labelling (ASL) with no vascular crushing (*i.e.* ASL noVC) (b), ASL with vascular crushing (c) and dynamic susceptibility contrast (DSC) (d) are shown with different scales. Arterial arrival time (AAT) images for ASL (e), ASL with vascular crushing (f) and delay time (DT) DSC (g) are shown with different scales. VC, vascular crushers.

**Figure 3.**
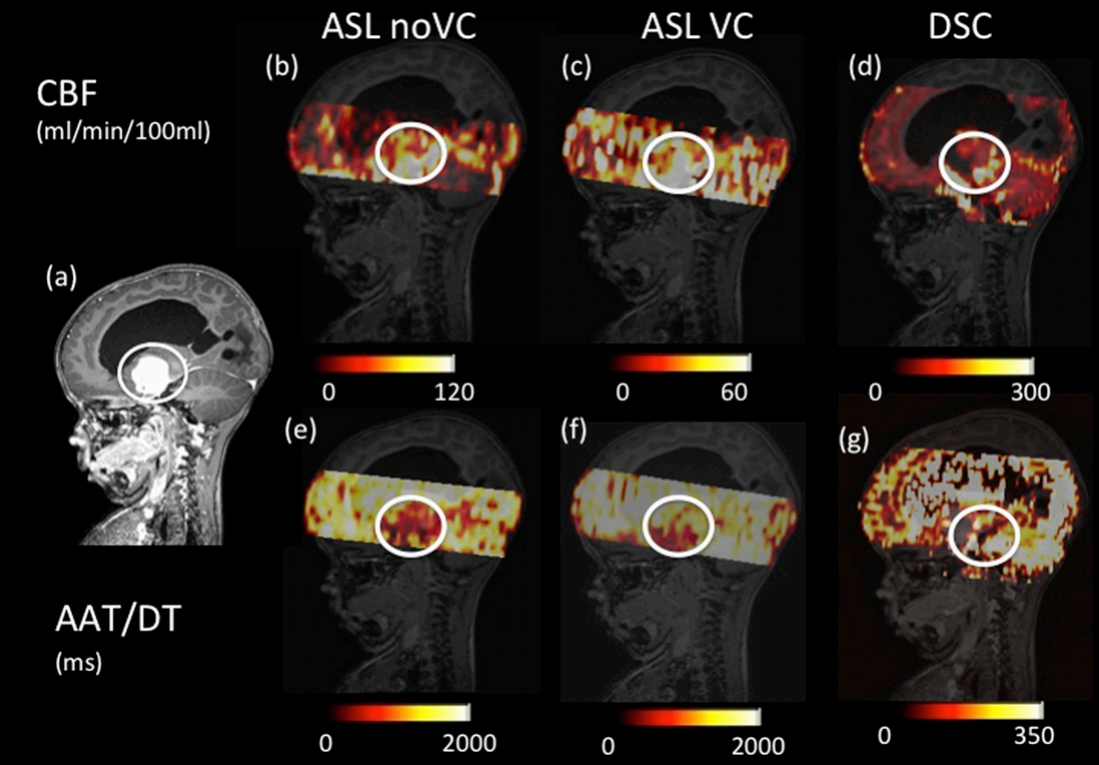
Axial images of a low-enhancing low-grade astrocytoma (highlighted in the white circles). (a) Shows a *T*_1_ weighted post-gadolinium image. Cerebral blood flow (CBF) images for arterial spin labelling (ASL) with no vascular crushing (*i.e.* ASL noVC) (b), ASL with vascular crushing (c) and dynamic susceptibility contrast (DSC) (d) are shown with different scales. Arterial arrival time (AAT) images for ASL (e), ASL with vascular crushing (f) and delay time (DT) DSC (g) are shown with different scales. VC, vascular crushers.

**Figure 4.**
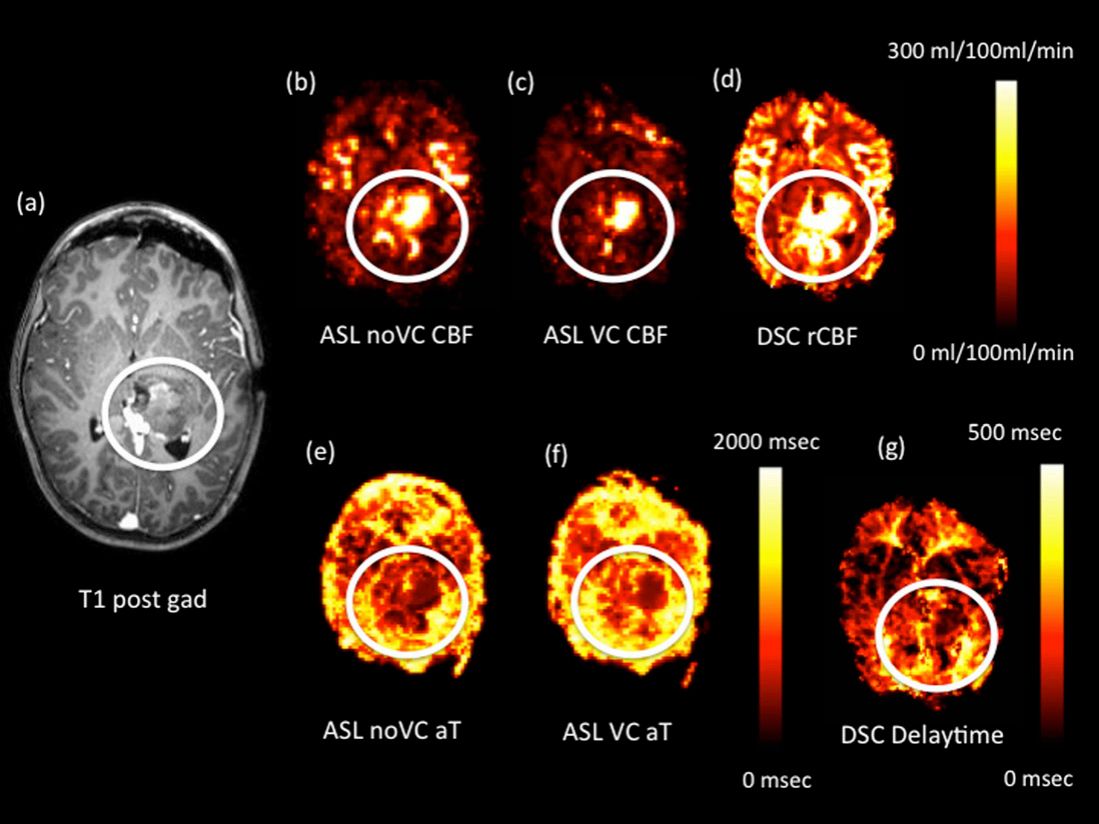
Intraoperative axial images from a single patient following a tumour-debulking surgery of a high-grade anaplastic astrocytoma (the region of residual tumour tissue is highlighted in the white circles). (a) Axial *T*_1_ weighted post-gadolinium (post gad) image. Cerebral blood flow (CBF) images for arterial spin labelling (ASL) with no vascular crushing (*i.e.* ASL noVC) (b), ASL with vascular crushing (c) and relative cerebral blood flow (rCBF) dynamic susceptibility contrast (DSC) (d) are shown with different scales. Arterial arrival time (aT) ASL with vascular crushing/delay time images for ASL (e), ASL with vascular crushing (f) and delay time from DSC (g) are shown with different scales. VC, vascular crushers.

Figures 2 and 3 show example quantitative output images from two patients, which show some similarities in spatial features in the corresponding ASL and DSC images, but also differences. For example, Figure 2 shows increased heterogeneity in the ASL images within the tumour region compared with the DSC maps. The ASL AAT images are particularly striking in this example, showing an increase in the arrival time within the tumour region in contrast to the other cases presented here (*i.e.* Figures 3 and 4).

An interesting case is shown in Figure 3 for a suprasellar glioma that was given a grade 1 classification, based on histopathological findings. Following surgical debulking, however, this tumour displayed aggressive biological behaviour and grew back quickly. The ASL data in particular show high blood flow in this tumour relative to the rest of the brain, suggesting that it is highly vascularized.

### Correlations between dynamic susceptibility contrast and arterial spin-labelling measures

Data from 20 patients were used to assess the significance of correlations between the perfusion metrics. Data from the single intraoperative example and the remaining three patients were not included, as they were found to have tumour blood flow values >±3 standard deviation from the mean and thus were found to be driving the correlations. In the tumour region, there is no significant correlation between any of the DSC and ASL measurements. This is in keeping with the descriptive statistics (Table1), which show higher mean blood flow within the tumour region than in the control region for DSC, but the opposite for ASL. In contrast, in the control region, significant correlations are observed between the DSC DT and ASL AAT (*r* = 0.77, *p* = 0.0002), in addition to the normalized measures of DSC rCBF and ASL CBF (*r* = 0.56, *p* = 0.02) (Table 2). Correlations are no longer significant for CBF measures when considering the vascular-crushed ASL measurements in comparison with DSC. There was, however, a significant correlation between the DSC DT and the ASL AAT_VC_ (r = 0.68, *p* = 0.003). In general, therefore, measurements are correlated in the control region but not in the tumour region, suggesting that the tumour pathology may be impacting the accuracy of modelling of the different MR measurements.

**Table 2.** Correlation coefficients (*r*) with accompanying significance (*p*) value

Control ROI correlations	*r*	*p*
DSC DT, ASL AAT	0.765	0.0002^*a*^
DSC rCBF, ASL CBF	0.561	0.015^*a*^
DSC DT, ASL AAT_VC_	0.685	0.0034^*a*^
DSC rCBF, ASL CBF_VC_	−0.003	0.992
DSC MTT, ASL AAT_VC_	0.185	0.493

AAT, arterial arrival time; AAT_VC_, arterial arrival time with vascular crusher; ASL, arterial spin labelling; CBF, cerebral blood flow; CBF_VC_, cerebral blood flow with vascular crusher; DSC, dynamic susceptibility contrast; DT, delay time; MTT, mean transit time; rCBF, relative cerebral blood flow; ROI, region of interest.

Correlations shown were obtained from the control ROI only, as tumour ROIs did not have any correlations that were significant between the DSC and ASL measures (not shown).

^*a*^ Significant correlations.

### Applications of dynamic susceptibility contrast and arterial spin labelling in an intraoperative example

Figure 4 shows an example from an intraoperative case of a high-grade anaplastic astrocytoma immediately following surgical debulking of the tumour. Clear spatial similarities are seen across all images, showing increased blood flow and reduced AAT/DT in the region where the residual tumour tissue is present (in the white circle).

Reductions in AAT and DT within the “tumour” region (Figure 4e,f and g) imply that blood arrives more quickly to these areas. High blood flow is also seen within this region on both the ASL (Figure 4b,c) and DSC (Figure 4d) CBF maps. Some of this may be due to bleeding; however, this would affect only the DSC CBF image, not the ASL image, which is largely insensitive to vessel leakage^22^ or blood pooling. The smaller region of elevated blood flow on the vascular-crushed ASL CBF image (Figure 4c) shows that some of the elevated “blood flow” in the other images is due to blood in large vessels. The vascular-crushed ASL allows true microvascular blood flow to be visualized, which may aid the surgeon in the detection of residual tumour, uncontaminated by large vessels or pooled blood.

## DISCUSSION

We have compared DSC and multi-timepoint ASL with and without VC in a small cohort of PBTs. We showed that DSC was overall quantitatively more sensitive to the tumour tissue, with significant differences in MTT and CBF between the tumour tissue and control (healthy) tissue, but not for the CBV measures. DSC DT measures were not different between the tumour and healthy tissue, and nor were any of the ASL output measures. It must be concluded therefore that DSC is more sensitive to the tumour tissue than ASL. The sensitivity of course assumes that the perfusion metrics change in the same way across this group of tumours. If, for example, true CBF goes up in some tumours and down in others, then significant changes across the group will be lost. Visually, it was noted that ASL maps showed a difference in the tumour region compared with the surrounding tissue in the majority of cases. There were, however, significant correlations between DSC and ASL output measures within the control region but not in the tumour region. The significant differences observed between tumour and control regions with DSC could be due to a number of reasons such as: (i) DSC measures are contaminated by “leaky” vasculature present in the tumour regions, thus impacting the overall measures and contributing to differences; (ii) AIF choice to model the perfusion profile of the contrast agent was acquired from healthy tissue regions close to the tumour and is not representative of the perfusion profile within the tissue. Therefore, AIF choice may be impacting the overall modelling of the DSC signal change in the tumour region, which is reflected by the significant differences; and finally, (iii) DSC could simply be more sensitive to the true perfusion differences within the tumour tissue.

Robust evidence supporting CBF differences in paediatric tumours as measured by DSC and ASL are sparse and it is only recently that ASL has been used in paediatric brain imaging^23^ and specifically in brain tumours.^1,24^ The strong correlations between CBF as measured with ASL and DSC within the control ROI (Table 2) are in line with other studies.^25–27^ The correlation was not significant for vascular-crushed ASL data, probably owing to the sensitivity of the vascular-crushed measures to small vessels, whereas DSC is dominated by larger vessels. DSC and ASL metrics were not correlated within the tumour region, supporting again the notion that tumour pathology may be impacting the accuracy of modelling, in particular the assumption of no contrast leakage in the case of DSC. There are, however, a number of studies that have compared DSC against ASL in adult brain tumours^28–30^ and other pathologies.^27,31^ Whilst it is not possible to compare the vascular characteristics of adult brain tumours with that of the paediatric population, it is interesting to observe that almost all of these studies observed strong correlations between normalized ASL CBF and DSC rCBF measures from within the tumour region. There was only one study^28^ that suggested that these correlations existed in a minority of the population studied. This discrepancy between the adult brain tumour literature and our data could be attributed to the pathophysiological differences in the PBTs and adult brain tumours.^32^

In this study, we considered the application of VC to the ASL sequence in order to sensitize the measurement to the smaller vessels. It can be observed that there is a general increase in the AAT measures with VC enabled compared with non-vascular-crushed data from both the tumour region as well as control region measures (Table 1). This is owing to blood taking longer to reach the microvascular bed.^33^ In addition, absolute CBF measures are lower with VC; this fits in with the expectation of lower blood flow in the small vessels, capturing true “perfusion”. The usefulness of ASL AAT maps with regard to visually identifying tumours is evident in Figures 2–4. These maps highlight the differences in the temporal dynamics of the blood flow within the tumour region when compared with the rest of the healthy brain. The relationship between AAT and tumour pathophysiology is not clear, but it could be speculated that the heterogeneity of AAT within the tumour region may relate to the differences in underlying tumour pathology. Visual comparisons of AAT maps with the DSC DT maps show a strong similarity, reflected in the significant correlation between these measurements in the control region. DSC DT measures in general have been used to correct the final output measures of CBF^34^ and differentiate between the penumbra and infarct core,^17^ however, on its own DSC DT measures have not been used clinically. MTT reflects the time it takes for blood to travel from the artery to the vein, which is generally altered in tumours owing to vessel shunts and tortuosity.^35^ A multi-timepoint ASL measure may be particularly beneficial over a single timepoint measurement in tumours, as the haemodynamics may be quite variable, making it unwise to assume a single fixed arrival time. Increasingly, a multi-timepoint ASL method is being used for clinical imaging; for example, in PBTs^8^ and adult patients with internal carotid artery occlusions.^9,36^

We used a relatively simple model to quantify CBF and AAT from our ASL data.^13^ A previous work in PBTs has used a more complex model, providing measures of pre-capillary and capillary arrival time.^8^ We felt that the lower temporal resolution of our ASL data (370 ms compared with 200 ms in Ref. 8,27) would not support a more complex model. However, our application of VC may achieve a similar result: AAT without crushers providing information similar to the pre-capillary arrival time, and AAT with crushers similar to the capillary arrival time. It is, however, difficult to validate this through direct comparisons between studies such as this study to that by Hales et al^8^ owing to differences in tumour types.

The results presented here support the feasibility of using multi-timepoint ASL to study the characteristics of blood flow within PBTs. The Look–Locker implementation of multi-timepoint ASL is time efficient, giving high-quality CBF and AAT images in only 4 min. This allowed for the application of the ASL technique in normal clinical practice as part of routine paediatric brain imaging. Our ASL CBF measures in the healthy control tissue fit well with the expected range of 29–80 ml/100 ml min^−1^, as reported by other ASL-based studies,^31,37^ including one study that incorporated a Look–Locker implementation. AAT measures reported in our work also fall within the range found in a separate study^38^ of 680–1066 ms. Standard deviation measures from our data do reflect a large range. This could be owing to the fact that measures were often derived from relatively small ROIs to match the tumour regions in some cases. The addition of VC in this work is a novel implementation that reduced the large-vessel signal, allowing different aspects of tumour vasculature to be measured, but also reduced the signal-to-noise ratio of the ASL technique further. Nevertheless, our intraoperative case suggested that this approach might help distinguish true tumour from large feeding vessels, which may be helpful to the surgeon. CBF measured with ASL and DSC is strongly correlated in the healthy brain, but not in the tumour tissue. This suggests that vessel leakage and altered vessel geometry is impacting the DSC measures. It is important to recognize that DSC was capable of differentiating tumour tissue through rCBF and MTT measures compared with ASL. ASL, however, may offer a more accurate measure of microvascular CBF that may be important in terms of diagnosis, treatment and prognosis.

## ACKNOWLEDGMENTS

We would like to acknowledge the support of Philips MR Clinical Science for the use of the prototype Vascular Crushing.

## FUNDING

This work was supported by the Medical Research Council Confidence in Concept scheme (grant ref number: MC_PC_12018) and Alder Hey Children's Charity.
